# Spontaneous recovery of effects of contrast adaptation without awareness

**DOI:** 10.3389/fpsyg.2015.01464

**Published:** 2015-09-30

**Authors:** Gaoxing Mei, Xue Dong, Bo Dong, Min Bao

**Affiliations:** ^1^Key Laboratory of Behavioral Science, Institute of Psychology, Chinese Academy of SciencesBeijing, China; ^2^Department of Psychology, Guizhou Normal UniversityGuiyang, China

**Keywords:** contrast adaptation, spontaneous recovery, awareness, visual memory, visual crowding, continuous flash suppression

## Abstract

Prolonged exposure to a high contrast stimulus reduces the neural sensitivity to subsequent similar patterns. Recent work has disclosed that contrast adaptation is controlled by multiple mechanisms operating over differing timescales. Adaptation to high contrast for a relatively longer period can be rapidly eliminated by adaptation to a lower contrast (or meanfield in the present study). Such rapid deadaptation presumably causes a short-term mechanism to signal for a sensitivity increase, canceling ongoing signals from long-term mechanisms. Once deadaptation ends, the short-term mechanism rapidly returns to baseline, and the slowly decaying effects in the long-term mechanisms reemerge, allowing the perceptual aftereffects to recover during continued testing. Although this spontaneous recovery effect is considered strong evidence supporting the multiple mechanisms theory, it remains controversial whether the effect is mainly driven by visual memory established during the initial longer-term adaptation period. To resolve this debate, we used a modified Continuous Flash Suppression (CFS) and visual crowding paradigms to render the adapting stimuli invisible, but still observed the spontaneous recovery phenomenon. These results exclude the possibility that spontaneous recovery found in the previous work was merely the consequence of explicit visual memory. Our findings also demonstrate that contrast adaptation, even at the unconscious processing levels, is controlled by multiple mechanisms.

## Introduction

The visual system is plastic, and can be shaped by experiences. Both longer-term experiences, e.g., perceptual learning (for a review, see Sasaki et al., 2010), and shorter-term experiences, e.g., visual adaptation (for reviews, Kohn, 2007; Wark et al., 2007; Webster, 2011), can alter neuronal sensitivity. Contrast adaptation refers to a phenomenon that prolonged exposure to high contrast stimuli increases perceptual thresholds of subsequent test stimuli (Blakemore and Campbell, 1969; Georgeson and Harris, 1984; Gardner et al., 2005; Kohn, 2007). Longer exposures to adapting stimuli have been found to generate stronger and more persistent contrast adaptation effects, which is referred to as duration scaling law (Greenlee et al., 1991; Bao and Engel, 2012).

To account for the temporal dynamics of contrast adaptation, one theory assumes that adaptation is controlled by a single neural mechanism operating at different timescales (Grzywacz and de Juan, 2003; Wark et al., 2009). However, the multiple mechanisms theory proposes that adaptation is controlled by multiple distinct mechanisms operating over differing timescales (Vul et al., 2008; Bao and Engel, 2012). A critical finding supporting the latter theory is the “spontaneous recovery” of adaptation effect observed in a few studies using “deadaptation” procedures (Vul et al., 2008; Bao and Engel, 2012; Bao et al., 2013; Mesik et al., 2013). In a deadaptation procedure, effects of longer period of adaptation are rapidly canceled by short exposures to adapters producing the opposite aftereffects (i.e., deadaptation). For example in Bao et al.' (2013) work, a baseline or neutral environment was first defined as prolonged adaptation to a medium contrast (25%) where effects of adaptation asymptoted. Adaptation to high contrast (80%) for a relatively longer period led to further loss of sensitivity. Subjects were then “deadapted” for a shorter period to a lower contrast (6.25%). The sensitivity loss caused by adaptation to high contrast stimuli were eliminated by the end of this deadaptation.

**Figure 1:**
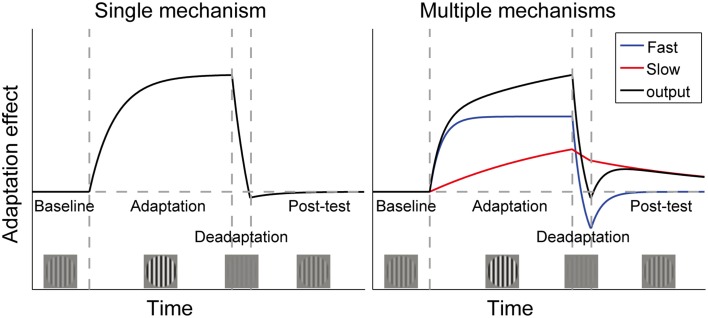
**Time courses of adaptation effects predicted by two theories of contrast adaptation**. Vertical dashed lines mark the onsets of adaptation (adapt to a contrast higher than baseline contrast), deadaptation (adapt to a contrast lower than baseline contrast), and a post-test (same as baseline contrast), respectively. If adaptation is controlled by a single neural mechanism, aftereffects will remain around the baseline level following deadaptation, which rapidly cancels the effect of the longer adaptation (left panel). However, the “spontaneous recovery” we found in our previous studies could not be interpreted by the single mechanism theory. It indicates that adaptation is controlled by multiple mechanisms with different time constants. For simplicity, the right panel only showed one fast and one slow mechanism. The black curve represents the sum of outputs of both mechanisms. Adaptation followed by rapid deadaptation may cause opposing signals from the two mechanisms. Decay in the post-test will affect the fast mechanism more strongly, leading to spontaneous recovery of adaptation due to the slow mechanism. (Bao et al., 2013). Copyright is held by the Association for Research in Vision and Ophthalmology (ARVO ©).

The pivotal question is what will happen if the visual system is again put into the neutral environment (25% contrast). As shown in Figure 1, the single mechanism theory predicts that the perceptual aftereffects of adaptation will remain around the baseline level following deadaptation, because the neuronal gain has already been adjusted to accommodate the neutral environment. Instead, the multiple mechanisms theory assumes that sensitivity is proportional to the sum of outputs of multiple controllers, with each operating over its own preferred timescale. The longer adaptation to high contrast causes both the long- and short-term mechanisms to signal for a sensitivity decrease, but the brief deadaptation may cause the short-term mechanisms to signal a sensitivity increase, canceling the effects of long-term adaptation. If the visual system is then put into the neutral environment, the short-term mechanisms will decay to baseline rapidly, allowing gradual unmasking of the original longer-term adaptation effect. This phenomenon is called “spontaneous recovery,” which has been repeatedly observed in studies using the “deadaptation” procedures (Vul et al., 2008; Bao and Engel, 2012; Bao et al., 2013; Mesik et al., 2013). A single mechanism cannot account for this recovery.

However, it remains an unresolved debate whether spontaneous recovery merely reflects the role of visual memory or not in these studies (Vul et al., 2008; Bao et al., 2013; Mesik et al., 2013), since subjects could always consciously see the adapters during the original adaptation period. This plausible account indeed urges an answer, given a recent report that visual short-term memory maintenance may increase the magnitude of tilt aftereffect (TAE) when the orientation of memory cue is consistent with the visual adapter (Saad and Silvanto, 2013).

Memory can be divided into explicit memory and implicit memory (Tulving, 1995). As a first step to explore the role of visual memory, the present study examined whether spontaneous recovery could be still observed when the adapting stimuli were removed from awareness. Rendering the adapters invisible can minimize the contribution from explicit visual memory. If spontaneous recovery disappears with this manipulation, visual memory should play a determinative role in the previous work. Taking advantage of the Continuous Flash Suppression (CFS) (Fang and He, 2005; Tsuchiya and Koch, 2005; Jiang et al., 2006; Faivre et al., 2014) and crowding techniques (Bouma, 1970; Toet and Levi, 1992; He et al., 1996; Greenwood et al., 2010; Whitney and Levi, 2011; Nandy and Tjan, 2012; Faivre et al., 2014), the two experiments in the present study concordantly witnessed the spontaneous recovery of effects of adaptation to invisible stimuli. Our results rule out the argument that explicit visual memory alone leads to spontaneous recovery observed in the “deadaptation” paradigms, and provide the first evidence that contrast adaptation can be controlled by multiple mechanisms even at the unconscious levels.

## Experiment 1: continuous flash suppression

### Methods

#### Participants

Breakthrough ratios of CFS were measured in 77 volunteers with a screen test (see Procedure for details). Here, breakthrough means that the invisible adapters overcome the interocular suppression by CFS stimuli and break into awareness. We selected participants with low breakthrough ratios in order to best separate the effects of invisible adapters on adaptation timecourse. A trial would be called a breakthrough trial if breakthrough occurred during its top-up period. While breakthrough ratio was referred to as the proportion of breakthrough trials in the screen test. Eventually, only 16 completed the formal experiment (8 females, ages ranging from 19 to 33 years, mean age: 22.1 years, see Results for more details about the selection of subjects). All had normal or corrected-to-normal vision, and were naïve to the purpose of the study except an author. Subjects were given informed consent prior to participation. Experimental procedures in the present study were approved by the Institutional Review Board of the Institute of Psychology, Chinese Academy of Sciences, and the work was carried out in accordance with the Code of Ethics of the World Medical Association.

#### Apparatus

Stimuli were generated in MATLAB (The MathWorks, Natick, MA) using PsychToolbox-3 (Brainard, 1997; Pelli, 1997) and were displayed on a 21-in. Dell Trinitron P1130 monitor (1024 × 768 pixels; 85 Hz refresh rate; gamma-corrected; mean luminance: 40 cd/m^2^). The display was driven by a Bits# 14-bit video card (Cambridge Research Systems), and was calibrated using a Photo Research PR-655 spectrophotometer. Subjects viewed the stimuli through a mirror stereoscope with a mounted chin rest at a distance of 100 cm in a dark room.

#### Stimuli

**Figure 2:**
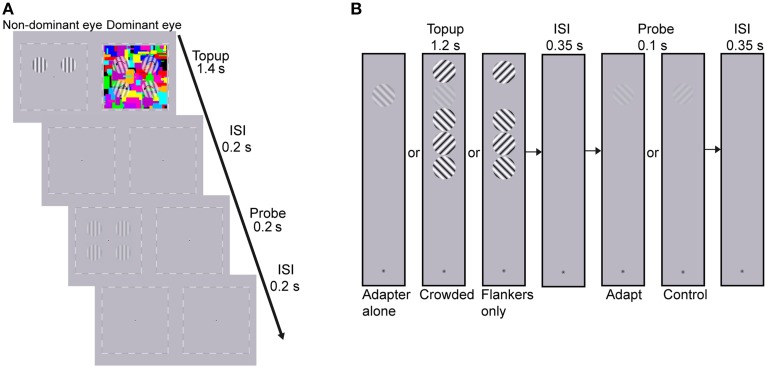
**Procedure of (A) the CFS condition of Experiment 1 and (B) Experiment 2. (A)** The dynamic CFS stimuli with four translucent rotating gratings were presented to the dominant eye which produced sufficiently low breakthrough ratio for the other eye in the screen test. One pair of drifting adapters was presented to the other eye for 1.4 s in each trial. After a 0.2 s blank inter-stimulus interval (ISI), the probes were presented for 0.2 s. Subjects were instructed to judge whether the contrasts of probes at the adapted locations were higher or lower than those at the unadapted locations. **(B)** Experiment 2 included three conditions: adapter alone, crowded and flankers-only conditions. Subjects adapted to the top-up stimulus for 1.2 s, followed by a 0.35 s blank ISI. Then the probe grating, oriented either 45 or 135°, was presented for 0.1 s. Subjects judged whether they perceived the probe grating (yes-no detection). The orientation of the target adapter, which was the second grating from the top, kept constant throughout a session while the orientation of the flanker nearest to the fixation point was randomly selected to be either 45 or 135° in each trial, with the constraint that neighboring flankers should be orthogonal to each other.

The adapters and probes were vertically oriented sinusoidal gratings with a spatial frequency of 2 cpd, whose edges were smoothed with a Gaussian filter. The gratings subtended 2° and were presented on the diagonal of four quadrants centered 2° away from the central fixation (see Figure 2A). The adapters were two high contrast gratings that were presented on either the upper or lower quadrants. The adapting locations were constant for each subject but were counter-balanced across subjects. To avoid afterimage and minimize the possibility of stimulus-driven eye movements during adaptation, the adapters drifted either toward or away from each other at the frequency of 1.43 Hz, with the drifting direction changing every 0.7 s. The probes were four static gratings that were presented at the four quadrants. The spatial phases of the probes were randomized across trials. But for ease of matching, the probes in the upper visual field remained in phase with those in the lower visual field.

The CFS stimuli (8 × 8°, flashing at 10 Hz) consisted of 60 Mondrian patterned images created by drawing rectangles of random colors and sizes. Since the CFS stimuli covered all the four testing locations, adaptation led by the CFS stimuli was believed to produce equal impact on the apparent contrast of probes among the four testing locations, as was also proved by a pilot test. To further strengthen interocular suppression, four translucent gratings rotating at 2.36 Hz were superimposed on the CFS stimuli at the four testing locations. Their initial orientations were random in each trial with the following constraints. The initial orientations were symmetric along both the horizontal and vertical meridians so that they could be grouped like a discontinuous diamond or X. The rotating directions were randomized across trials but also remained symmetric along both meridians. For example in the upper right, upper left, lower left, and lower right visual field, the rotating direction of the grating could be ccw, cw, ccw, and cw. The sizes of the rotating gratings were identical to the adapters presented to the non-dominant eye (see Figure 2A). A black-and- white square frame (8.5 × 8.5°) and a central fixation point were always presented binocularly to help fusion.

#### Procedure

Similar to the previous studies (Vul et al., 2008; Bao et al., 2013; Mesik et al., 2013), the present work adopted a top-up paradigm. Test contrasts were tracked at the adapted locations, which were matched to a constant contrast level at the unadapted locations with staircases. The timecourse of the test contrasts revealed the dynamic of contrast adaptation. The adapters could be either visible or rendered invisible by means of the CFS technique. To delineate the timecourse of adaptation effects to invisible adapters, it was desired to have as few breakthrough trials as possible. The time needed for suppressed stimuli to break into awareness (i.e., breakthrough time) has been found to vary across subjects with stable inter-individual differences (Sklar and Hassin, 2015). Since the adapters and CFS stimuli were displayed for only 1.4 s per trial in our experiment, longer breakthrough time would correspond to lower breakthrough ratio. Therefore, we screened subjects with a preliminary test before running the formal experiment. Those whose breakthrough ratio for at least one eye was lower than 5% were likely to proceed to the formal experiment where the adapters were always presented to the eye with lower breakthrough ratio (termed non-dominant eye).

In the screen test, the CFS stimuli and adapters (80% contrast) were presented dichoptically during the top-up adaptation for 1400 ms, followed by a 200 ms interval and four probes (30% contrast) which were presented for 200 ms. Subjects were asked to press the space bar once they saw any part of the adapters. The eye of origin of the stimuli was randomly determined every 50 s. Each session lasted for 400 s, and subjects had to finish two sessions of screen tests.

Once passing the screen test, subjects were required to complete three stages of practices (usually two sessions per stage). Each practice session at the first stage lasted for 150 s where there were no adapters or CFS stimuli but only probes for contrast matching that were presented either binocularly or only to the non-dominant eye. The goal of this stage was to make subjects familiar with the contrast matching task in a top-up paradigm. Stimuli at the second stage included both probes and adapters that were presented to the non-dominant eye only. Each session consisted of two 150-s phases. Top-up adapters (30% contrast) were only displayed in the second phase. This allowed us to evaluate whether subjects showed typical adaptation effects to the baseline adapting contrast (30%). Subjects without showing clear adaptation effects here would be excluded from the subsequent experiments, because this suggests that either the matching performance was too noisy or the adaptation effects were too small to be reliably measured given our used stepsize of staircases. Both might impede the observation of clear timecourses of adaptation effects. At the third stage, CFS stimuli were presented to the dominant eye as top-ups, allowing the subjects to get used to the CFS top-up presentations. No adapters were presented in this stage to avoid potential training effects on adaptation.

There were three conditions in the formal experiment, binocular, CFS, and monocular condition. The binocular condition was basically a procedural replication of the contrast matching experiment in our previous work (Bao et al., 2013), where subjects viewed the adapters and probes binocularly throughout a session. In the CFS condition, the CFS stimuli and adapters were viewed dichoptically, with the adapters and probes always presented to the non-dominant eye. The monocular condition served as a control condition. The procedure was the same as that in the CFS condition except that there were no CFS stimuli.

Each trial started with two drifting adapters which were presented for 1400 ms. The contrast of the adapters ramped up to its highest level within the initial 500 ms and faded out in the last 100 ms. After a 200 ms interval, four probes simultaneously presented for 200 ms. The contrast of each probe varied with a Gaussian temporal profile that had a standard deviation of 50 ms. After another 200 ms interval, the next trial started. Subjects were required to judge whether the probes at the adapted locations appeared to have higher or lower contrast than those at the unadapted locations by pressing one of two keys. The contrast of the probes at the unadapted locations was constant at 30%, while the contrast of the probes at the adapted locations was updated by a one-down-one-up staircase with a starting contrast of 30%. The initial step size of the staircase was 10% contrast. It decreased to 6% after three reversals, and dropped to 2% after another three reversals.

In the CFS condition, besides the contrast matching task, subjects were also required to press the space bar whenever they perceived any part of the adapters. Once this happened, the CFS stimuli and adapters would be removed from the screen. The time that each breakthrough occurred was recorded. This allowed us to run a replay session after each CFS session, where top-up adapters were presented binocularly just in the corresponding trials where breakthroughs occurred in the previous CFS session. In the rest of trials of the replay sessions, only the CFS stimuli were presented. The “replay” sessions helped evaluate the influence of the few breakthrough trials on the time course of adaptation effects. If visual memory rather than the long adaptation is the main cause for spontaneous recovery, one may speculate that seeing the adapters infrequently throughout the long adaptation period could still form similar memory representations of the adapters like in the no-CFS sessions (monocular or binocular conditions), e.g., subjects might use mental imagery to strengthen the memory representations during the CFS-only trials. This might also lead to a spontaneous recovery. If spontaneous recovery is drived by long adaptation, no spontaneous recovery should be observed in the replay sessions. After all, it is not very likely for those infrequent brief adaptations to activate the long-term mechanisms.

**Figure 3:**
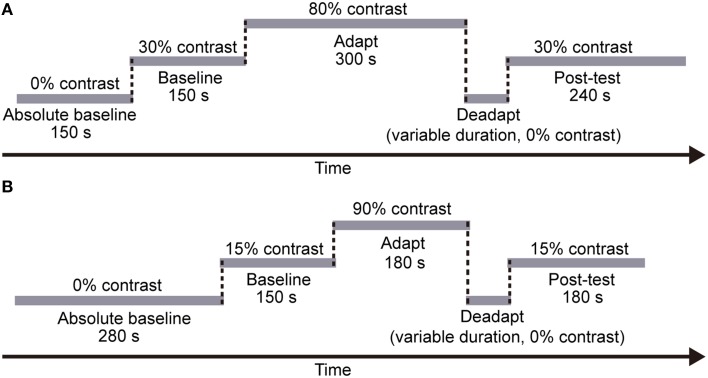
**The procedure of each session in (A) Experiment 1 and (B) Experiment 2, respectively**. In both experiments, each session included five stages: an “absolute baseline” period with no adapters, a “baseline” period with medium contrast adapters, an “adaptation” period with high contrast adapters, a “deadaptation” period with no adapters, and a “post-test” period again with medium contrast adapters. Note that the duration of each stage was fixed across subjects and sessions. However, the deadaptation duration varied across sessions (see Procedure for details).

Each session included five stages (see Figure 3A): a 150-s “absolute baseline” period where no adapters were presented, a 150-s “baseline” period with medium contrast (30%) adapters, a 5-min “adaptation” period with high contrast (80%) adapters, a “deadaptation” period with no adapters (i.e., meanfield), and a 240-s “post-test” period again with medium contrast (30%) adapters. According to our pilot data, effects of adaptation decayed more rapidly when using meanfield for “deadaptation,” especially at the periphery. Therefore, we chose meanfield in the present study. Deadaptation was terminated whenever the test contrast was lower than the baseline (see Analysis for how we estimated baselines) or the duration of deadaptation reached 120 s. Each subject finished eight sessions for each condition with a counter-balanced session sequence. Subjects had to take a break in the normal visual environment for at least 1 h before the next session started.

#### Analysis

For each subject, the test contrasts of the last 15 reversals in the baseline period were averaged to estimate the baseline effect of adaptation in each session. This baseline was then subtracted from the entire timeseries for normalization. Figure S3 in the Supplementary Materials showed the raw data of two sessions from one subject. The timeseries before and after deadaptation were nearest-neighbor interpolated to a 2 second sample interval and then averaged across sessions, respectively. To evaluate whether there was a significant adaptation effect after exposure to high contrast gratings, the averaged test contrast of the last 5 reversals in the adaptation period was compared with zero using paired *t*-test. A linear trend analysis was also performed on the test contrasts from the beginning of the post-test to the peak time (obtained by inspecting the grand average timecourse for each condition) to examine the occurrence of spontaneous recovery. Breakthrough ratios in the formal experiments were calculated only for the 5-min adaptation period, where the adapting contrast was as high as in the screen test.

### Results

In 23 subjects, breakthrough ratios for both eyes exceeded the screening criterion (5%), therefore they were not allowed to participate in the subsequent experiments. Among the 54 subjects who passed the screen test, 8 quit on their own wills without starting to practice. Thirteen did not finish the practice because 3 quit and 10 failed to show reliable adaptation effects during the practice sessions (see Procedure for details). Another 17 subjects finished the practice but were not asked to complete the formal experiment, because during deadaptation their effects of adaptation failed to decay to baselines within 120 s. Eventually, 16 subjects completed the entire experiment, whose results were thus reported below.

**Figure 4:**
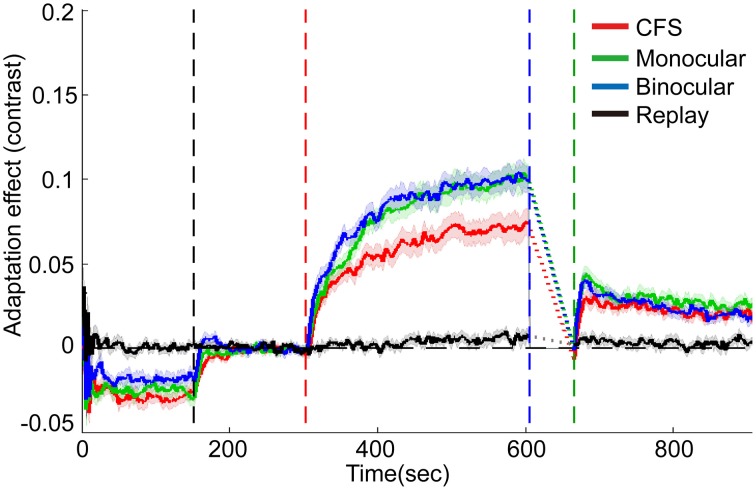
**Grand average timecourses for three conditions and replay sessions in Experiment 1**. Shaded regions show ±1 s.e of the mean. Because of variable deadaptation duration across conditions, timecourses during deadaptation were not plotted but substituted by dashed lines. Adaptation effects were normalized by subtracting average matching contrasts of the last 15 reversals in the baseline period. The horizontal dashed lines represent the normalized baselines. The four vertical dashed lines denote the start time for the baseline, adaptation, deadaptation, and post-test period, respectively.

The effects of contrast adaptation were observed in all the three conditions, as revealed by the increased mean test contrasts of the last 5 reversals in the adaptation period [see Figure 4, CFS condition: *t*_(15)_ = 8.96, *p* < 0.001, monocular condition: *t*_(15)_ = 13.08, *p* < 0.001, binocular condition: *t*_(15)_ = 13.26, *p* < 0.001]. The magnitude of such asymptotic effects of adaptation for the CFS, monocular and binocular condition was 7.1, 10.0, and 10.0%, respectively (See the individual data plots in Figures S1, S2 in the Supplementary Materials). A One-Way ANOVA showed a main effect of conditions [*F*_(2, 45)_ = 4.70, *p* < 0.05]. The *post-hoc* paired comparison *t*-tests with Bonferroni method revealed that the adaptation effects were weaker in the CFS condition than in the monocular and binocular conditions (*p* < 0.05 for both comparisons).

During deadaptation, the adaptation effects rapidly decayed to the baseline levels in all the conditions (deadaptation duration: CFS, 40.9 ± 17.0 s; monocular, 72.0 ± 17.0 s; binocular, 67.9 ± 16.9 s). The linear trend analysis showed that reliable spontaneous recovery emerged rapidly in the initial phase of the post-test for all the conditions [CFS, 0-30 s, *t*_(15)_ = 5.75, *p* < 0.001; monocular, 0-18 s*, t*_(15)_ = 6.12, *p* < 0.001; and binocular, 0-18 s, *t*_(15)_ = 6.55, *p* < 0.001]. Then adaptation effects decayed slowly but still remained above the baseline by the end of the post-tests (*p* < 0.05 for all time points).

In most subjects, the breakthrough ratio was kept very low (only 2% on average, ranging from 0 to 6% for 15 subjects, 17% for one subject). The analysis on the replay condition suggested that the infrequent brief perception of the adapters failed to produce noticeable adaptation effects [see Figure 4, about 0.7% for the last 5 reversals in the adaptation period, *t*_(15)_ = 2.03, *p*>0.05].

## Experiment 2: visual crowding

### Methods

#### Participants

Fifteen subjects participated in Experiment 2 (8 females, ages ranging from 20 to 33 years, mean age: 22.5 years). Three of them had participated in Experiment 1.

#### Apparatus

Same apparatuses were used as in Experiment 1, except that the viewing distance was 53 cm.

#### Stimuli

All stimuli were sinusoidal gratings oriented at 45 or 135°. The spatial frequency of the gratings was 2 cpd. The target adapter and flankers subtended 2.1°, and the probe subtended 1.9°. The target adapter and probe were centered 17° above the fixation point which located 12.9° below the screen center.

The layout of the stimuli in the crowded condition was similar to that in He et al.'s (1996) work. The target adapter and four flankers were aligned at the vertical meridian, with a center to center distance of 2.2° (see Figure 2B). The target adapter located at the second from the top. The orientation of the flanker nearest to the fixation was randomly selected from the two candidate orientations (45 and 135°) in each trial. The adjacent flankers were perpendicular to each other. There were two control conditions: adapter alone, and flankers only conditions.

#### Procedure

Subjects were required to finish two sessions of pre-test. Since all of them performed at a chance level in reporting the orientation of the adapter, they were all permitted to participate in the formal experiment. In each trial of the pre-test, the adapter and flankers were presented for 1.2 s, with a 1-s blank inter-trial interval. The orientation of the adapter was randomly selected to be either 45 or 135° in each trial. Subjects were asked to indicate the orientation of adapter. There were 300 trials in each session of pre-test. To confirm crowding effect was present throughout the experiment, subjects finished another two such sessions after the formal experiment.

Like the CFS experiment, in the formal experiment, each session included five stages (see Figure 3B), a 280-s “absolute baseline” period (no adapter), a 150-s “baseline” period (15% contrast adapter), a 180-s “adaptation” period (90% contrast adapter), a “deadaptation” period without adapter, and a 180-s “post-test” period (15% contrast adapter). The duration of deadaptation was determined in the same way as that in Experiment 1 except that the maximum duration was cut down to 72 s because of the shorter “adaptation” period. The contrast of the flankers was always 90%.

In each trial, the top-up stimuli lasted for 1.2 s. After a 350-ms blank interval, a probe was presented for 100 ms. The next trial started after another 350 ms. Subjects adapted to a constant orientation (either 45 or 135°) in a session, and the two adapting orientations were counter-balanced across sessions. The orientation of the probe was randomly selected to be either 45 or 135°, but evenly distributed over time through a session. The subjects judged whether the test grating can be perceived or not by key presses (yes-no paradigm).

A one-down-one-up staircase procedure was used to track the contrast detection thresholds. For the crowded and adapter-alone conditions, subjects finished six sessions for each adapting orientation. Subjects also finished six sessions for the flankers-only condition.

#### Analysis

The analysis of Experiment 2 was similar to that in Experiment 1 except the followings. Average detection thresholds of the last 10 reversals in the absolute baseline stage and in the baseline stage served as the estimations of absolute baselines and baselines, respectively. To reduce the inter-individual variance on detection thresholds, the timecourse of contrast threshold was divided by the absolute baseline. To evaluate the influences from the flankers on the thresholds, for the flankers-only condition we conducted a linear regression analysis on each subject's average timecourse after the start of the baseline stage. Based on the slopes of the fits, the timecourses for the crowded condition were then linear detrended. To estimate the new baseline for the detrended timecourses, we first calculated the mean duration corresponding to the last 10 reversals of the baseline stage for the crowded condition in each session. The normalized thresholds within this mean duration were then averaged to serve as the new baseline for each subject.

### Results

**Figure 5:**
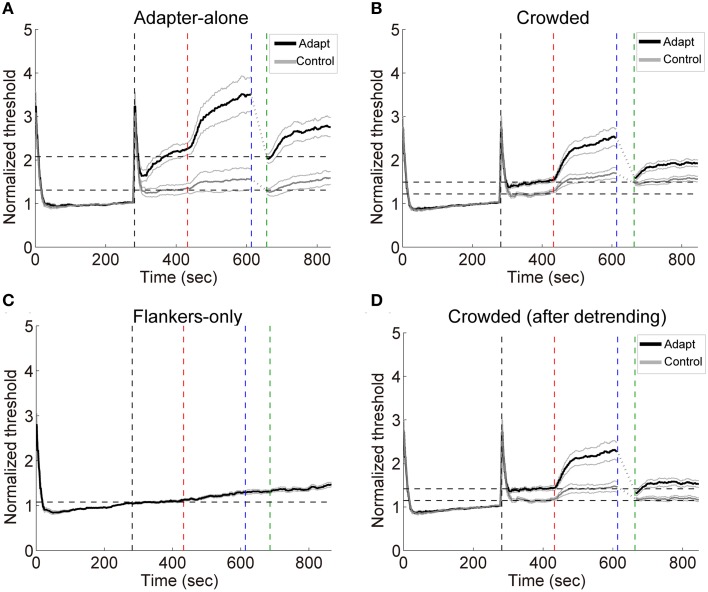
**(A–C)** Grand average timecourses in Experiment 2. Thick black and gray curves represent the timecourses for the adapting and control orientations, respectively. Normalized thresholds were calculated by dividing the timecourse by the absolute baseline. The horizontal dashed lines represent the normalized baselines. **(D)** The timecourses for the crowded condition. The influences from the flankers were estimated with linear regression on the flankers-only data. The slopes of the fits were used to calculate the linear trend in the timecourses for the crowded condition led by the flankers. The black and gray curves here show the detrended timecourses of thresholds. The four vertical dashed lines mark the start of the baseline, adaptation, deadaptation and post-test period, respectively.

Subjects performed the orientation identification task at the chance level before and after the formal experiment (accuracy: 50.6 ± 3.1% and 51.2 ± 3.3%, respectively. Also see Table S1 in the Supplementary Materials for a list of individual accuracies), suggesting that they failed to discriminate the orientation of adapters in the crowded condition. The result patterns for the 7 subjects who performed slightly “better” than chance mostly resembled those for the other 8 subjects (See Figures S4, S5 in the Supplementary Materials). Exposure to high contrast gratings significantly elevated the detection thresholds at the target location for the adapting orientation [see Figures 5A,B, crowded: *t*_(14)_ = 5.65, *p* < 0.001; adapter-alone: *t*_(14)_ = 4.07, *p* < 0.01]. The adaptation effects were orientation specific, which were significantly stronger for the adapting orientation than for the control orientation [crowded: *t*_(14)_ = 4.40, *p* < 0.001; adapter-alone: *t*_(14)_ = 6.01, *p* < 0.001].

During deadptation, the adaptation effects returned to the baseline in the adapter-alone and crowded conditions (deadaptation duration: crowded, 49.9 ± 9.1 s; adapter-alone, 43.3 ± 12.8 s). Spontaneous recovery was also found in both the adapter-alone, [*t*_(14)_ = 5.96, *p* < 0.001] and crowded [*t*_(14)_ = 9.70, *p* < 0.001] conditions. In the flankers-only condition, we noticed a gradual ascending trend of the contrast thresholds (Figure 5C). The ascending trend was perhaps caused by adaptation to flankers, fatigue or other factors. To remove the trend, we ran a linear regression to fit each subject's averaged timecourses for the flankers-only condition. The linear regression model predicted 97% of the total variance of the data. Then we took the slopes of the fits to calculate the linear trend contributed from the flankers. After the detrending, a spontaneous recovery was still observed in the beginning of post-test [Figure 5D, *t*_(14)_ = 5.00, *p* < 0.001]. After the recovery, adaptation effects remained above the baseline (*p* < 0.05 for all time points).

## Discussion

Using the CFS and visual crowding methods, we observed spontaneous recovery of effects of contrast adaptation to unaware stimuli. Because unconscious processing of the adapters is not thought to help establish and reinforce explicit memory of the adapters, this evidence suggests that explicit visual memory is not a main cause of spontaneous recovery of contrast adaptation.

The spontaneous recovery phenomenon has been historically investigated in Pavlovian conditioning (for reviews, see Bouton, 1993; Rescorla, 2004), where the conditioned responding can return spontaneously after a period of time following extinction. A constellation of evidence has proposed that memory and memory retrieval play a critical role in modulating spontaneous recovery in classical conditioning (for a review, see Bouton and Moody, 2004). Our previous work disclosing spontaneous recovery in visual adaptation is methodologically inspired by classical conditioning literatures. However, the findings in the present study suggest that the spontaneous recovery phenomena observed in the two fields should be based on dissociated neural mechanisms.

One open question is whether implicit memory for unconscious processing of the adapters contributes to spontaneous recovery of contrast adaptation. However, a recent study on implicit memory hints that the answer to this question is probably no (Yang et al., 2011). The authors found that exposure of subliminal facial stimulus by backward masking generated significant priming effects only when the invisible primes were fearful faces rather than neutral faces. This indicates that only unconscious processing on emotional stimulus can form implicit memory. The adapters in the present study were gratings, which were obviously non-emotional. Although they should receive sufficient visual processing to produce sizeable effects of adaptation, it is very unlikely that such unconscious processing can form implicit memory potentially responsible for spontaneous recovery. To strictly address this issue, future work may consider applying the deadaptation paradigms on patients with impaired perceptual implicit memory (Gong et al., 2015). Another possibility is to use classical conditioning to induce a valence into adapters of one orientation, and compare the timecourses of aftereffects led by the adapters with and without the induced valence.

The present study also introduced a modified CFS paradigm that effectively removed high contrast adapters from awareness for almost the entire 5 min of adaptation. The modified CFS stimuli were created with the popular “Mondrian” CFS stimuli superimposed with rotating gratings of the same spatial frequency as the adapters. These superimposed gratings probably made the CFS stimuli more closely resemble the adapters in the Fourier amplitude spectrum, thus helped further lower the breakthrough ratio (Yang and Blake, 2012). This was indeed demonstrated by one of our pilot tests [breakthrough ratio: modified CFS (33.6%) vs. classical CFS (49.7%), *t*_(4)_ = 3.55, *p* < 0.05]. Unlike previous reports of unreduced adaptation effects from invisible stimuli of high contrast (Blake et al., 2006), we observed weaker effects of adaptation to high contrast gratings in the CFS condition than in the monocular and binocular conditions. This discrepancy may reflect stronger suppression generated by CFS stimuli than other binocular rivalry stimuli (Tsuchiya and Koch, 2005). Stronger suppression of adapters was believed to decrease the neuronal activity in the visual cortex as early as in V1 (Yuval-Greenberg and Heeger, 2013; but see Watanabe et al., 2011). Accordingly, it was not surprising that the effects of adaptation were attenuated in the CFS condition.

It should be warned that although the subjects were instructed to press a key as long as they saw any piece of the adapters in the CFS experiment, it cannot fully be excluded that they failed to report breakthroughs and that the selection of subjects by the screen test might actually bias toward observers that are less likely to report a breakthrough. We adopted the present method for screening lots of volunteers quickly. An ideal procedure, however, would display the adapters of one of the two orientations randomly and force the subjects to report the orientation of the adapters, whether the adapters are visible or not. A qualified subject should perform this screen test at a chance level. Future work will examine the suppression efficiency of our modified CFS method with more objective measurements in larger samples.

In summary, the present study, by virtue of the techniques of CFS and crowding, for the first time demonstrates that multiple controlling mechanisms for contrast adaptation can operate even at the unconscious levels. Since the effects of adaptation we measured were elicited by adaptation to unseen stimuli, our observations of spontaneous recovery in the unconscious conditions exclude the possibility that spontaneous recovery of contrast adaptation are dominated by mediation from explicit visual memory.

## Author contributions

GM, XD, BD, and MB designed the research. GM, XD performed the research. GM and MB analyzed the data. GM, XD, and MB wrote the paper.

### Conflict of interest statement

The authors declare that the research was conducted in the absence of any commercial or financial relationships that could be construed as a potential conflict of interest.
