# Supplementary material for: Spontaneous recovery of effects of contrast adaptation without awareness
Source: Front Psychol. 2015 Sep 30;6:1464. doi: 10.3389/fpsyg.2015.01464 (PMC4588121; doi:10.3389/fpsyg.2015.01464)
Supplement: Supplementary file 1 [file DataSheet1.DOCX]

**Supplementary materials**

Gaoxing Mei, Xue Dong, Bo Dong, Min Bao


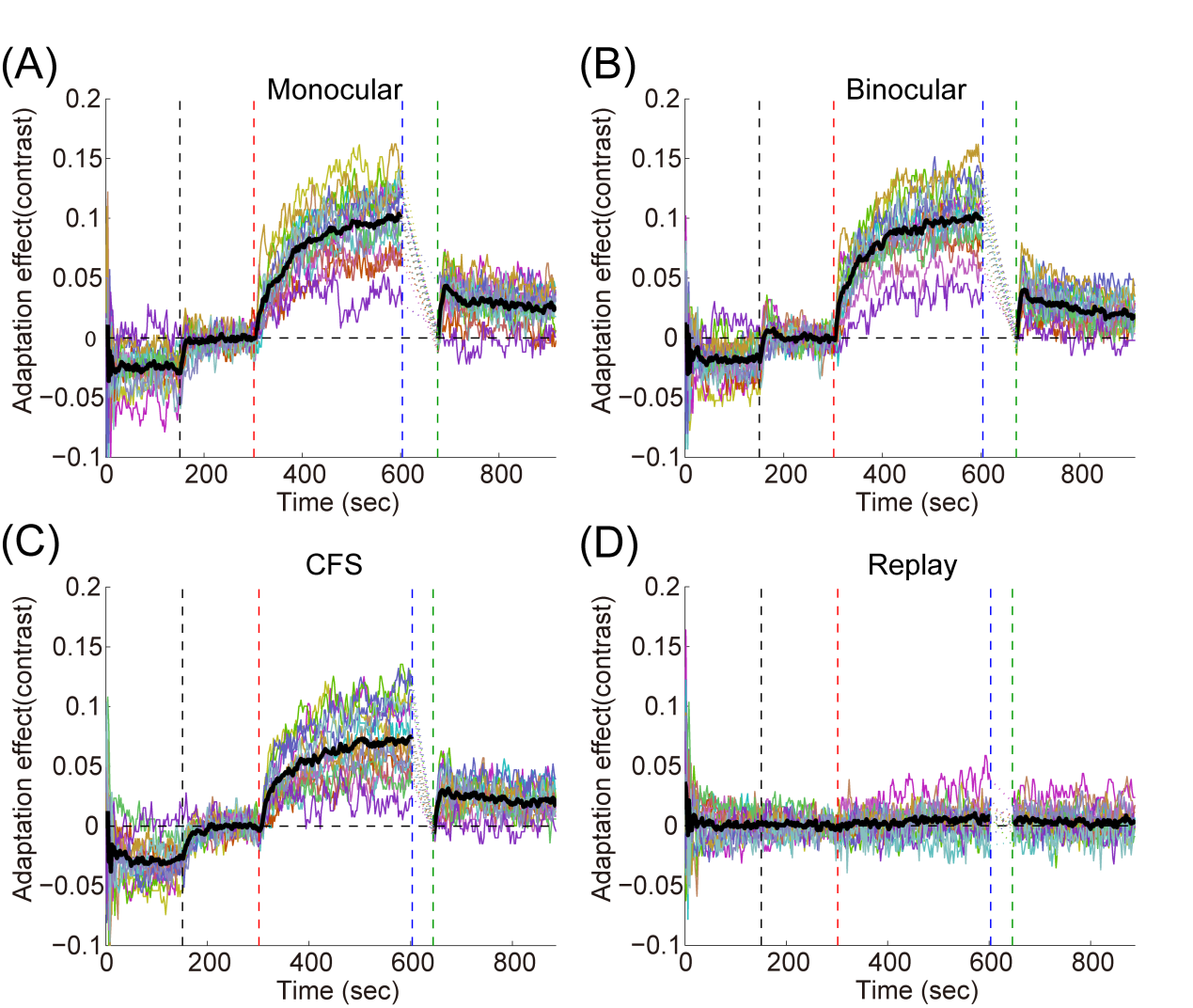


**Figure S1**. Grand average (black thick) and individual (colorful) timecourses in Experiment 1.


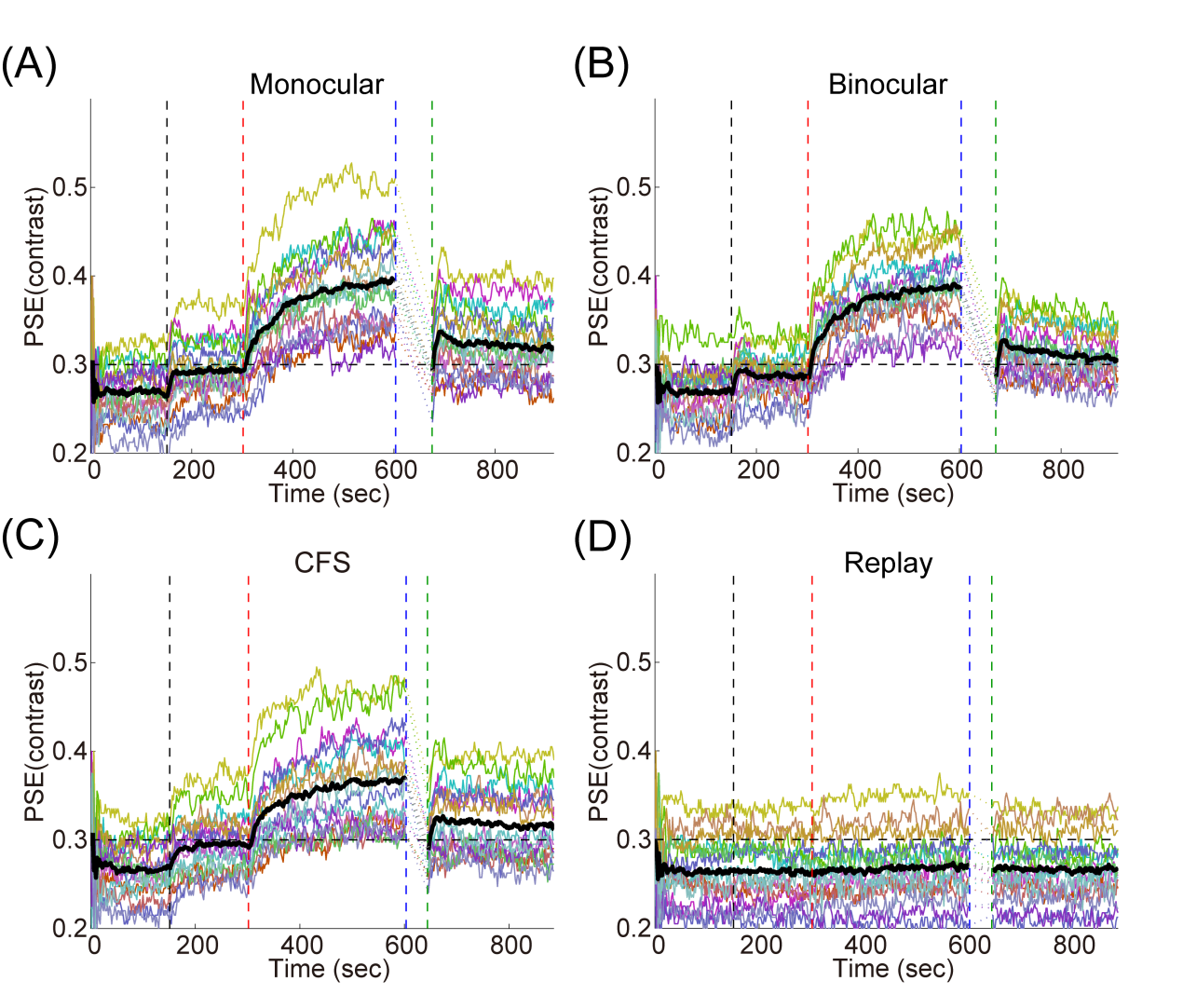


**Figure S2**. Grand average and individual timecourses in Experiment 1 plotted with the PSE contrast as the y-axis. The horizontal lines denote 30% contrast.


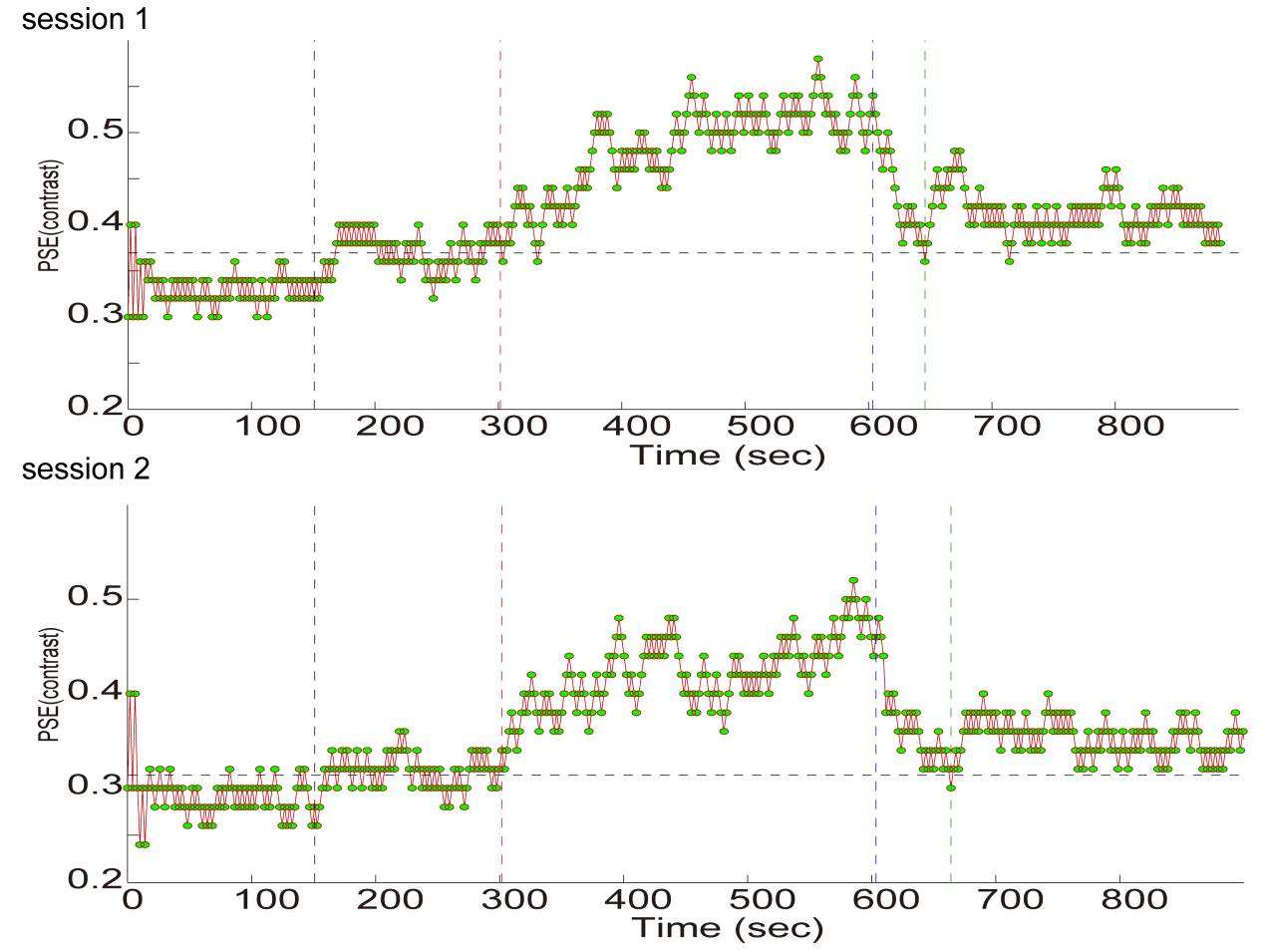


**Figure S3**. Raw data of two sessions from one subject before the nearest-neighbor interpolation.

**Table S1**. Performance of the orientation identification task in Experiment 2

| Subject | Pretest | Post test | Average |
| --- | --- | --- | --- |
| S1 | 0.4833 | 0.4633 | 0.4733 |
| S2 | 0.4483 | 0.53 | 0.4892 |
| S3 | 0.51 | 0.4733 | 0.4917 |
| S4 | 0.5433 | 0.5417 | 0.5425 |
| S5 | 0.5617 | 0.5367 | 0.5492 |
| S6 | 0.5333 | 0.52 | 0.5267 |
| S7 | 0.4983 | 0.5133 | 0.5058 |
| S8 | 0.5 | 0.55 | 0.525 |
| S9 | 0.5 | 0.495 | 0.4975 |
| S10 | 0.495 | 0.4917 | 0.4933 |
| S11 | 0.5467 | 0.525 | 0.5358 |
| S12 | 0.4667 | 0.4433 | 0.455 |
| S13 | 0.485 | 0.54 | 0.5125 |
| S14 | 0.4983 | 0.555 | 0.5267 |
| S15  Average | 0.525  0.5063 | 0.5  0.5119 | 0.5125  0.5091 |


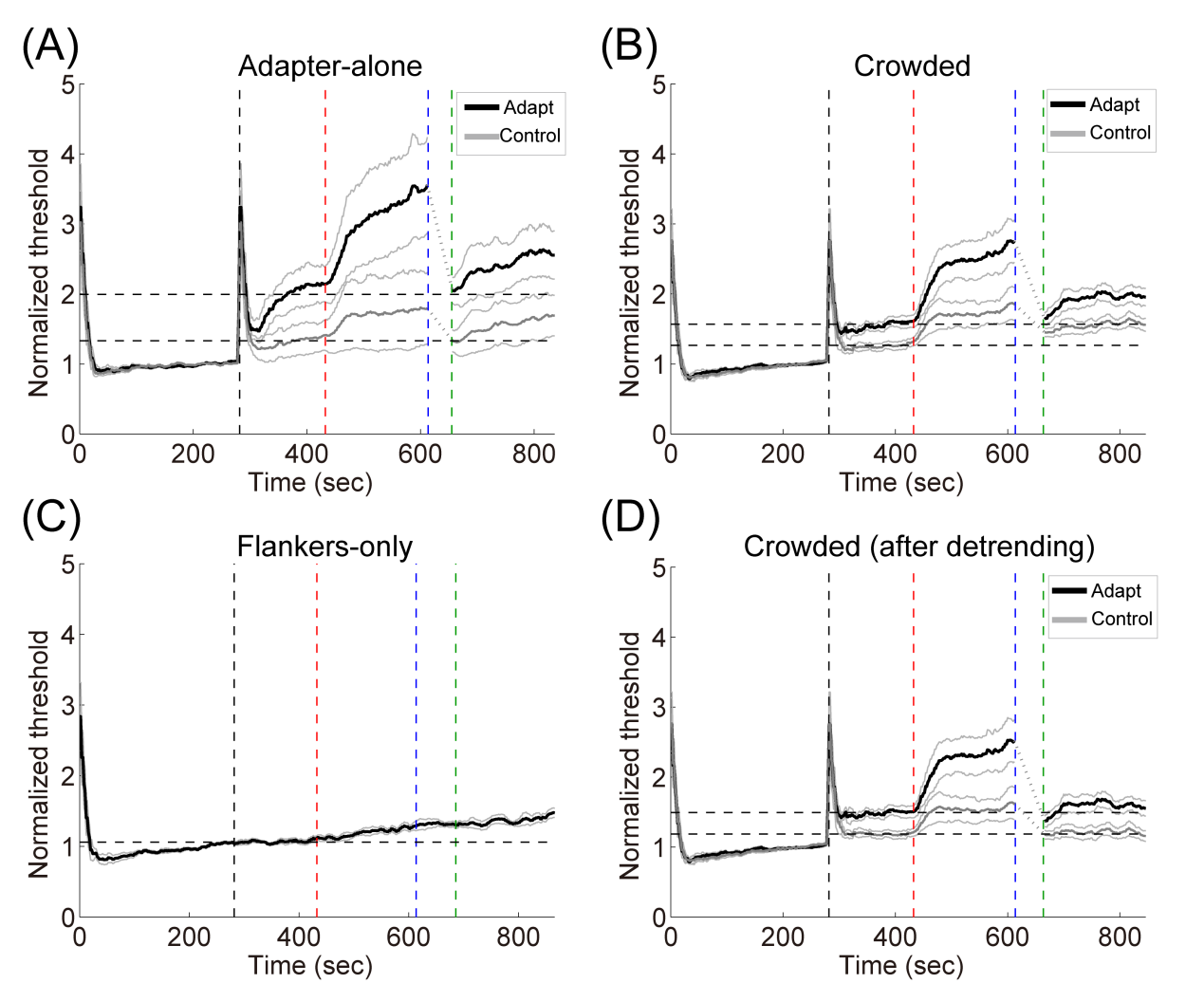


**Figure S4**. Grand average timecouses for the subjects whose performances of the orientation identification task were between 48% and 52% (7 subjects).


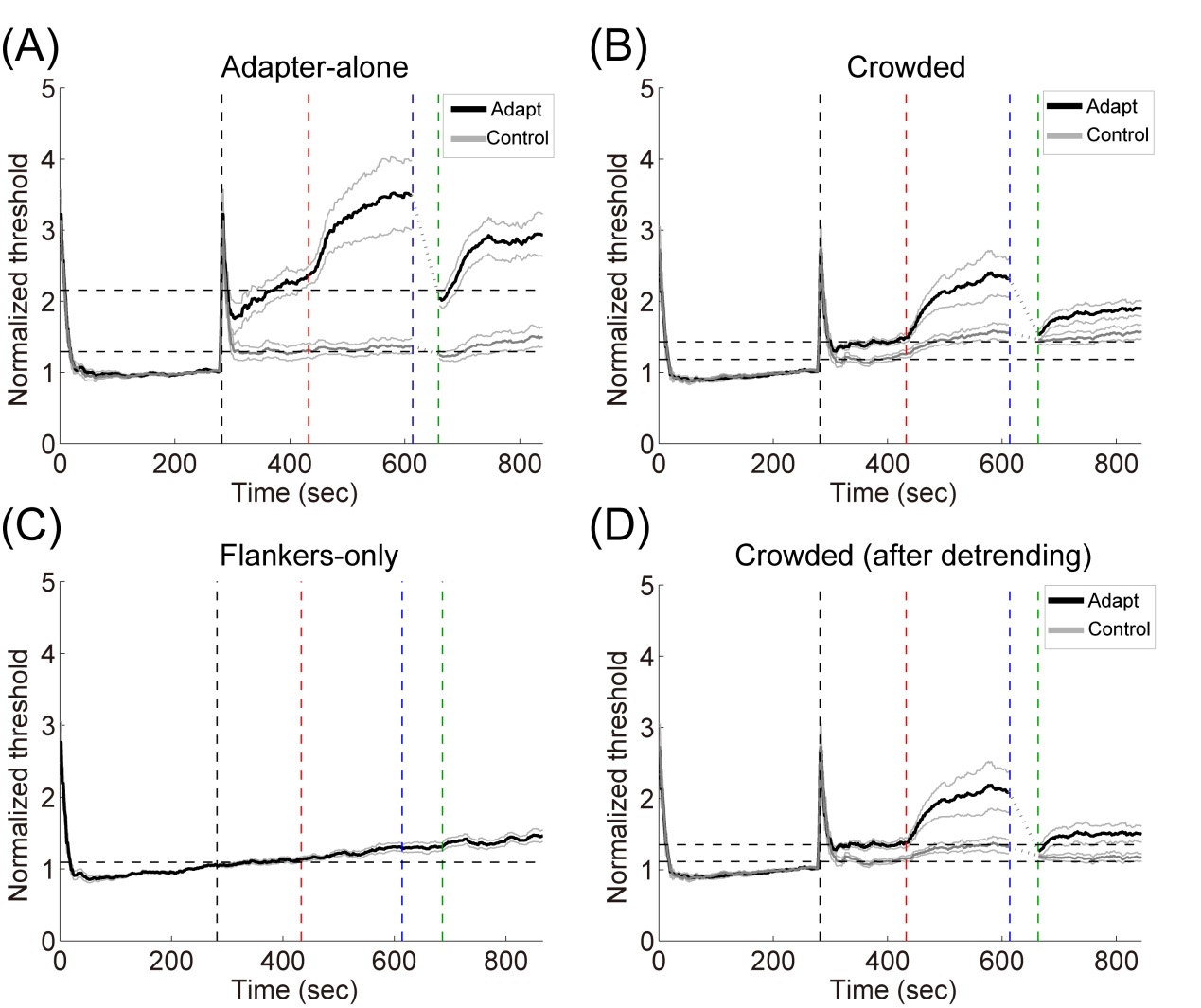


**Figure S5**. Grand average timecouses for the subjects whose performances of the orientation identification task were below 48% or above 52% (8 subjects).

**Table S2**. The ratios of the average test contrasts of the last 5 reversals in the adaptation period between the adapting and control orientation in Experiment 2

| Subject | adapter-alone condition | crowded condition |
| --- | --- | --- |
| S1 | 2.7724 | 1.6482 |
| S2 | 1.2797 | 1.327 |
| S3 | 1.6619 | 1.124 |
| S4 | 3.8933 | 2.1952 |
| S5 | 2.941 | 1.4543 |
| S6 | 2.2975 | 1.4001 |
| S7 | 2.6608 | 1.7318 |
| S8 | 1.6188 | 0.9894 |
| S9 | 1.8054 | 1.3252 |
| S10 | 2.9233 | 2.055 |
| S11 | 1.5224 | 1.121 |
| S12 | 1.5754 | 1.3166 |
| S13 | 1.4905 | 1.1046 |
| S14 | 3.5979 | 2.0302 |
| S15 | 2.5106 | 1.5801 |
| mean±SD | 2.3034±0.8177 | 1.4935±0.3744 |

After normalization by dividing each column by its mean, the mean for each column become 1, and the SDs are 0.3550 (adapter-alone) and 0.2507 (crowded).

**Table S3**: Breakthrough ratio in the screen test

|  | Dominant eye (%) | Nondominant eye (%) |
| --- | --- | --- |
| Subjects who completed the entire experiment | 13±18 | 2±2 |
| Subjects who failed to pass the screen test | 38±21 | 24±14 |

**Table S4**: Subjects list

Note: Breakthrough ratios in the formal experiments were calculated only for the 5-min adaptation period, where the adapting contrast was as high as in the screen test. The subject No. does not represent the temporal order of participation in the study. To protect the subjects’ privacy, their names were removed in this form.

| Ss #1-16 (N = 16) completed the entire experiment. Note that after screening lots of subjects, we allowed two subjects (#1 and #25) to proceed to the formal experiment. The breakthrough ratios for their weaker eyes were higher than 5%, but still lower than 10%. Eventually, only subject #1 completed the entire experiment. | | | | | | | | | |
| --- | --- | --- | --- | --- | --- | --- | --- | --- | --- |
| Subject No. | Breakthrough ratio  (screen test) | | | Numbers of formal sessions completed | | | breakthrough ratio in the completed sessions | | Note |
|  | Stronger eye | Weaker eye | | CFS | monocular | binocular |  |  |  |
| 1 | 0.65 | 0.06 | | 8 | 8 | 8 | 0.1733 | | Author G.M. |
| 2 | 0.18 | 0.005 | | 8 | 8 | 8 | 0.0592 | |  |
| 3 | 0.04 | 0.03 | | 8 | 8 | 8 | 0.0125 | |  |
| 4 | 0.01 | 0 | | 8 | 8 | 8 | 0 | |  |
| 5 | 0.375 | 0 | | 8 | 8 | 8 | 0 | |  |
| 6 | 0.065 | 0.025 | | 8 | 8 | 8 | 0 | |  |
| 7 | 0.005 | 0.005 | | 8 | 8 | 8 | 0.0133 | |  |
| 8 | 0 | 0 | | 8 | 8 | 8 | 0 | |  |
| 9 | 0.055 | 0.005 | | 8 | 8 | 8 | 0 | |  |
| 10 | 0.315 | 0.04 | | 8 | 8 | 8 | 0.0033 | |  |
| 11 | 0.05 | 0.045 | | 8 | 8 | 8 | 0.0083 | |  |
| 12 | 0.005 | 0 | | 8 | 8 | 8 | 0 | |  |
| 13 | 0.065 | 0.04 | | 8 | 8 | 8 | 0.0033 | |  |
| 14 | 0.065 | 0.05 | | 8 | 8 | 8 | 0.0517 | |  |
| 15 | 0 | 0 | | 8 | 8 | 8 | 0 | |  |
| 16 | 0.18 | 0 | | 8 | 8 | 8 | 0.0042 | |  |
| Though passing the screen test, Ss #17-33 (N = 17) did not complete the experiment because their data were found to either *fail to decay to the baseline*s (FDB) within the maximum deadaptation duration that we permitted (120 s) or showed unwanted *high breakthrough ratio* (HBR). Here ‘quit’ means that the subject requested to quit the experiment usually because of time conflict or feeling uncomfortable with the stimuli. | | | | | | | | | |
| Subject No. | Breakthrough ratio  (screen test) | | Numbers of formal sessions completed | | | | | breakthrough ratio in the completed sessions | Note |
|  | Stronger eye | Weaker eye | CFS | | monocular | binocular | |  |  |
| 17 | 0.01 | 0.0067 | 2 | | 3 | 3 | | 0.01 | FDB |
| 18 | 0.2133 | 0.0367 | 2 | | 1 | 1 | | 0.19 | HBR |
| 19 | 0 | 0 | 2 | | 1 | 1 | | 0.2333 | HBR |
| 20 | 0 | 0 | 2 | | 1 | 1 | | 0.0767 | FDB |
| 21 | 0.1 | 0.005 | 1 | | 0 | 0 | | 0.2533 | HBR |
| 22 | 0.005 | 0 | 2 | | 0 | 0 | | 0.1267 | HBR |
| 23 | 0 | 0 | 1 | | 0 | 0 | | 0 | quit |
| 24 | 0.01 | 0.005 | 2 | | 2 | 3 | | 0 | FDB |
| 25 | 0.305 | 0.095 | 1 | | 1 | 1 | | 0.2 | HBR |
| 26 | 0.66 | 0.04 | 3 | | 1 | 1 | | 0.1689 | HBR |
| 27 | 0.2667 | 0.0033 | 1 | | 1 | 1 | | 0.0067 | FDB |
| 28 | 0 | 0 | 3 | | 3 | 3 | | 0 | FDB |
| 29 | 0 | 0 | 2 | | 3 | 1 | | 0 | FDB |
| 30 | 0 | 0 | 1 | | 1 | 0 | | 0.9 | HBR |
| 31 | 0.425 | 0.02 | 1 | | 2 | 3 | | 0 | FDB |
| 32 | 0.69 | 0.015 | 2 | | 0 | 0 | | 0.73 | HBR |
| 33 | 0.59 | 0.005 | 0 | | 1 | 1 | | - | quit |
| Ss #34-56 (N = 23) failed to pass the screen test and did not proceed into the formal sessions. | | | | | | | | | |
| Subject No. | Breakthrough ratio  (screen test) | | | Numbers of formal sessions completed | | | breakthrough ratio in the completed sessions | | Note |
|  | Stronger eye | Weaker eye | | CFS | monocular | binocular |  |  |  |
| 34 | 0.39 | 0.35 | | - | - | - | - | | HBR |
| 35 | 0.3767 | 0.06 | | - | - | - | - | | HBR |
| 36 | 0.25 | 0.23 | | - | - | - | - | | HBR |
| 37 | 0.305 | 0.19 | | - | - | - | - | | HBR |
| 38 | 0.46 | 0.43 | | - | - | - | - | | HBR |
| 39 | 0.0767 | 0.07 | | - | - | - | - | | HBR |
| 40 | 0.33 | 0.32 | | - | - | - | - | | HBR |
| 41 | 0.385 | 0.185 | | - | - | - | - | | HBR |
| 42 | 0.235 | 0.1 | | - | - | - | - | | HBR |
| 43 | 0.44 | 0.33 | | - | - | - | - | | HBR |
| 44 | 0.32 | 0.15 | | - | - | - | - | | HBR |
| 45 | 0.13 | 0.0625 | | - | - | - | - | | HBR |
| 46 | 0.62 | 0.335 | | - | - | - | - | | HBR |
| 47 | 0.1867 | 0.13 | | - | - | - | - | | HBR |
| 48 | 0.33 | 0.17 | | - | - | - | - | | HBR |
| 49 | 0.83 | 0.45 | | - | - | - | - | | HBR |
| 50 | 0.265 | 0.245 | | - | - | - | - | | HBR |
| 51 | 0.6 | 0.4 | | - | - | - | - | | HBR |
| 52 | 0.28 | 0.12 | | - | - | - | - | | HBR |
| 53 | 0.15 | 0.105 | | - | - | - | - | | HBR |
| 54 | 0.3833 | 0.3033 | | - | - | - | - | | HBR |
| 55 | 0.935 | 0.55 | | - | - | - | - | | HBR |
| 56 | 0.35 | 0.195 | | - | - | - | - | | HBR |
| Ss # 57-64（N =8）passed the screen test, but they felt uncomfortable with the stimuli. Therefore they did not start the practice, let alone the formal sessions. | | | | | | | | | |
| Subject No. | Breakthrough ratio  (screen test) | | | Numbers of formal sessions completed | | | breakthrough ratio in the completed sessions | | Note |
|  | Stronger eye | Weaker eye | | CFS | monocular | binocular |  |  |  |
| 57 | 0 | 0 | | - | - | - | - | | quit |
| 58 | 0 | 0 | | - | - | - | - | | quit |
| 59 | 0 | 0 | | - | - | - | - | | quit |
| 60 | 0.005 | 0 | | - | - | - | - | | quit |
| 61 | 0.02 | 0.01 | | - | - | - | - | | quit |
| 62 | 0 | 0 | | - | - | - | - | | quit |
| 63 | 0.19 | 0.005 | | - | - | - | - | | quit |
| 64 | 0.015 | 0 | | - | - | - | - | | quit |
| Ss #65-77 (N = 13) passed the screen test, and then started the practice, but did not proceed into the formal experiment, because most of them failed to show typical adaptation effect (NAE) to 30% contrast adapters during the practice sessions. | | | | | | | | | |
| Subject No. | Breakthrough ratio  (screen test) | | | Numbers of formal sessions completed | | | breakthrough ratio in the completed sessions | | Note |
|  | Stronger eye | Weaker eye | | CFS | monocular | binocular |  |  |  |
| 65 | 0 | 0 | | - | - | - | - | | NAE |
| 66 | 0 | 0 | | - | - | - | - | | NAE |
| 67 | 0 | 0 | | - | - | - | - | | NAE |
| 68 | 0.22 | 0.02 | | - | - | - | - | | NAE |
| 69 | 0.1067 | 0.0467 | | - | - | - | - | | quit |
| 70 | 0.0367 | 0.03 | | - | - | - | - | | quit |
| 71 | 0.025 | 0.01 | | - | - | - | - | | NAE |
| 72 | 0.01 | 0 | | - | - | - | - | | NAE |
| 73 | 0.005 | 0 | | - | - | - | - | | NAE |
| 74 | 0.0267 | 0.0067 | | - | - | - | - | | NAE |
| 75 | 0.11 | 0 | | - | - | - | - | | NAE |
| 76 | 0 | 0 | | - | - | - | - | | quit |
| 77 | 0.075 | 0.025 | | - | - | - | - | | NAE |
